# Host plant defense signaling in response to a coevolved herbivore combats introduced herbivore attack

**DOI:** 10.1002/ece3.224

**Published:** 2012-05

**Authors:** Anastasia M Woodard, Gary N Ervin, Travis D Marsico

**Affiliations:** 1 Department of Biological Sciences, Arkansas State UniversityP.O. Box 599, State University, Arkansas, 72467; 2 Department of Biological Sciences, Mississippi State UniversityP.O. Box GY, 295 Lee Blvd., Mississippi State, Mississippi, 39762

**Keywords:** Coevolution, defense priming, herbivore-induced plant volatiles, HIPVs, insect herbivore, invasive species, naïve host, plant defense, plant–plant signaling

## Abstract

Defense-free space resulting from coevolutionarily naïve host plants recently has been implicated as a factor facilitating invasion success of some insect species. Host plants, however, may not be entirely defenseless against novel herbivore threats. Volatile chemical-mediated defense signaling, which allows plants to mount specific, rapid, and intense responses, may play a role in systems experiencing novel threats. Here we investigate defense responses of host plants to a native and exotic herbivore and show that (1) host plants defend more effectively against the coevolved herbivore, (2) plants can be induced to defend against a newly-associated herbivore when in proximity to plants actively defending against the coevolved species, and (3) these defenses affect larval performance. These findings highlight the importance of coevolved herbivore-specific defenses and suggest that naïveté or defense limitations can be overcome via defense signaling. Determining how these findings apply across various host–herbivore systems is critical to understand mechanisms of successful herbivore invasion.

## Introduction

From thorns to poison, plants have evolved a variety of mechanisms to combat herbivory. While some defenses are constitutive, others are induced only upon perception of attack to allow for optimal allocation of resources (Karban and Baldwin 1997; Agrawal 1998; Karban 2011). Inducible plant defenses rely heavily upon herbivore-induced plant volatiles (HIPVs) as a signaling mechanism both for initiating systemic defense response throughout the attacked plant, as well as for signaling predators and parasitoids of the herbivore as an indirect defense (Kessler and Baldwin 2001; Heil and Silva Bueno 2007; Dicke 2009; Dicke and Baldwin 2010; Wu and Baldwin 2010). In addition, it has been shown that HIPVs function as signals between neighboring plants, indicating an increased probability of attack and allowing for upregulation of defense pathways in unattacked plants (Baldwin and Schultz 1983; Engelberth et al. 2004; Peng et al. 2005; Arimura et al. 2010; Wu and Baldwin 2010; Karban 2011). This “primed state” (Prime–A–Plant Group 2006) allows plants to simultaneously avoid the costs of implementing defenses in the absence of enemies, while minimizing damage during the time required to mount defenses once an attack is initiated (Peng et al. 2005; van Hulten et al. 2006; Frost et al. 2008; Karban 2011).

Defense priming has been shown to result in a faster, more intense response, specifically tailored to the threatening herbivore (Frost et al. 2008) and, thus, represents an elegant example of mechanisms that can arise during the coevolutionary arms race between plants and their attackers (Kant and Baldwin 2007; Dicke and Baldwin 2010; Wu and Baldwin 2010). Research to date, however, has failed to examine the effects of defense priming or signaling in a system with a novel (i.e., introduced) threat (Kant and Baldwin 2007), despite the increasing number of such new associations globally. Here we investigate differential defense responses of host plants to a native versus an exotic insect herbivore and show that (1) host plants defend more effectively against the coevolved herbivore, (2) plants can be induced to defend against a newly-associated herbivore when in proximity to plants actively defending against the coevolved species, and (3) these defenses affect larval performance.

**Figure 1:**
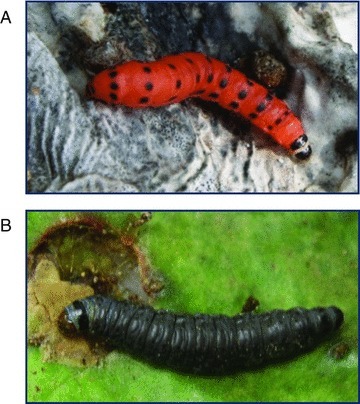
Fifth instar cactus-boring moth larvae. (A) *Cactoblastis cactorum* (Berg) and (B) *Melitara prodenialis* Walker.

We studied the cactophagous pyralids *Cactoblastis cactorum* (Berg) and *Melitara prodenialis* Walker, which are associated predominantly with pricklypear cacti of the genus *Opuntia* (Cactaceae). North America has 10 species (in two genera, *Melitara* and *Ozamia*) of native *Opuntia*-feeding pyralids (Neunzig 1997). In 1989, however, the historic biological control agent *C. cactorum*, a species native to Argentina and neighboring countries, was discovered to have colonized Florida (Habeck and Bennett 1990) (Fig. 1A). Since then, *C. cactorum* has spread across the coastal regions of the southeastern United States (Legaspi and Legaspi 2010; Madsen 2011), where it attacks all *Opuntia* species within this region (Solis et al. 2004; Simonson et al. 2005; Sauby 2009).

**Figure 2:**
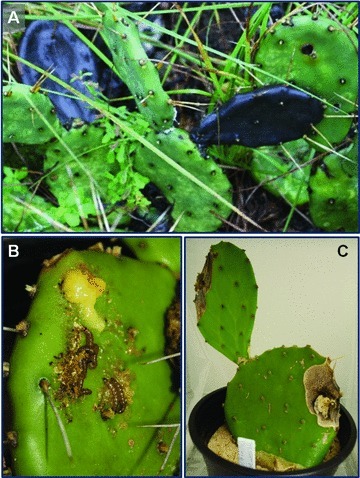
Observable inducible defenses of *Opuntia*. (A) Programmed cell death defense in *Opuntia humifusa*, (B) mucilage defense in *O. humifusa*, and (C) programmed cell death defense and mucilage in *Opuntia stricta*.

*Melitara prodenialis*, the only species of cactophagous moth to naturally inhabit the southeastern United States (Fig. 1B), does not commonly negatively impact growth of *Opuntia* individuals or populations (Carlton and Kring 1994; Baker and Stiling 2009), indicating that the host plants are able to tolerate *M. prodenialis* feeding. In the field and laboratory, *Opuntia humifusa* (Raf.) Raf. and *Opuntia stricta* (Haw.) Haw., the two most abundant host plants in the southeastern United States, have been observed defending against *M. prodenialis* by sectioning off cladodes via an apparent programmed cell death response, often within 48 h of larval feeding (personal observation), or by exuding thick mucilage (Mafokoane et al. 2007) (Fig. 2). We hypothesized that these defense responses decrease larval survivorship by deterring feeding and increasing opportunities for larval desiccation, predation, and parasitism. These easily observed induced plant defenses normally are not exhibited by North American *Opuntia* infested with *C. cactorum* (personal observation). We document here greater defense response of *Opuntia* to herbivory by the native coevolved herbivore *M. prodenialis* than to the newly-associated invasive herbivore *C. cactorum*. Our results are important for implicating defense-free space as a key factor that has facilitated the invasion of *C. cactorum* and allowed it to become a destructive pest, whereas *M. prodenialis* is not outbreaking. We also document the apparent communication of defenses from plants responding to the coevolved native *M. prodenialis* to plants experiencing attack by the newly-associated *C. cactorum*. We suggest HIPVs as the mechanism of this defense transfer. Our findings highlight the importance of coevolved herbivore-specific defenses and suggest that naïveté or defense limitations can be overcome via defense signaling.

## Materials and Methods

**Figure 3:**
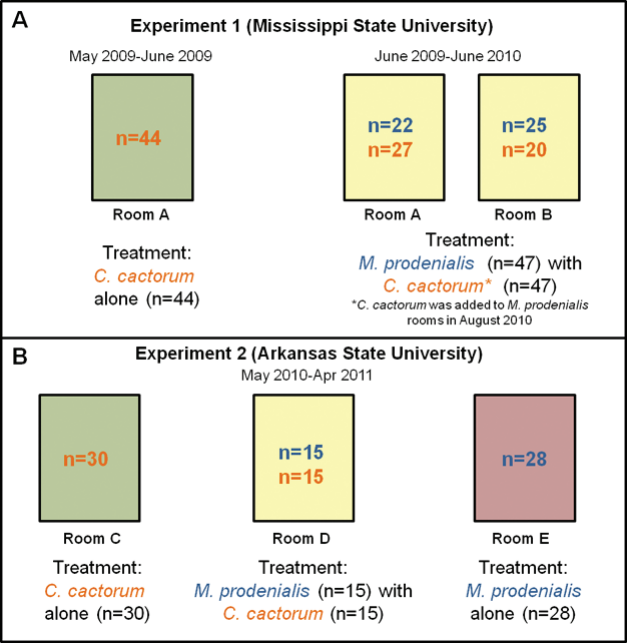
Experimental design of the two rearing experiments. (A) For Experiment 1 at Mississippi State University (2009–2010), two rearing rooms were used. The treatment of *Cactoblastis cactorum* reared only in the presence of plants infested with *C. cactorum* was separated in time from the treatment of *C. cactorum* reared in the presence of plants fed on by *Melitara prodenialis* (as well as *M. prodenialis* reared in the presence of plants fed on by *C. cactorum*). Half of the cages contained *Opuntia stricta* as the host plant, and the other half contained *Opuntia humifusa*. (B) In Experiment 2 at Arkansas State University (2010–2011), four treatments (*C. cactorum* reared only in the presence of plants fed on by *C. cactorum, C. cactorum* reared in the presence of plants fed on by with *M. prodenialis, M. prodenialis* reared in the presence of plants fed on by *C. cactorum*, and *M. prodenialis* reared only in the presence of plants fed on by *M. prodenialis*) were separated in space, in three rearing rooms. Half of the cages contained *O. stricta* as the host plant, and half contained *O. humifusa*.

Two replicate experiments were conducted in which larvae of the native (*M. prodenialis*) and invasive (*C. cactorum*) moths were reared separately on host plants housed within mesh cages. Each cage contained a single herbivore species, but treatments varied based on combinations of herbivore species in each rearing room as depicted in Figure 3. Plants and insects were wild collected from the Florida Panhandle, USA. Cladodes of *O. humifusa* and *O. stricta* were cut from large wild plants and planted in two-liter pots filled with sand collected from a dredge site in Northeast Mississippi, then allowed to root in a greenhouse for at least one season (e.g., February–April) before inclusion in the study. Immediately before the experiments, *C. cactorum* and *M. prodenialis* egg masses were collected from *Opuntia* spp. from field locations in the Florida Panhandle, USA, and transported back to USDA APHIS-approved quarantine facilities.

Each of the total 226 experimental cages began with a single, greenhouse-grown, potted *Opuntia* host plant and 10–20 eggs or neonate larvae of one of the herbivore species. In each treatment, equal numbers of *O. humifusa* and *O. stricta* were used as hosts for each moth species. Cages containing the different host species were intermingled within each room. Laboratory experiment conditions were held at 26°C, 12L:12D, and 50% or higher relative humidity. Rearing rooms contained 30 cm × 30 cm × 38 cm PVC-frame cages, each encased in a sewn polyester no-see-um mesh (∼100 holes per cm^2^) that was tied closed with nylon rope. Herbivore treatments included rooms containing each herbivore reared alone and rooms containing both herbivore species (Fig. 3). There was airflow within each rearing room facilitated by individual air handling units that was not recirculated but vented out of the building. Additional greenhouse-grown host plants were added as needed until larval feeding ceased. Plants were watered as needed every two to three weeks with 300 mL of tap water.

Entry into the host, feeding, and defense responses were recorded twice weekly for the duration of the experiments, and cages were inspected twice a week for the presence of pupae. All pupae were removed from their silken cocoons for weighing and sexing. Observed plant defenses were assigned a numerical value that ranged from no defense to severe defense: 0 = no defense, 1 = single drop of mucilage, 2 = thin mucilage, 3 = thick mucilage, and 4 = mucilage and programmed cell death. Most mortality directly from host plant defenses (e.g., larvae becoming trapped in mucilage) happened early, within the first month of the experiment, when larvae were small. Defenses continued through larval feeding, but necrosis from feeding damage became increasingly hard to differentiate from programmed cell death defense. Thus data are only presented on defenses that occurred during the first month of larval feeding. Plant defenses were analyzed for all plants using a Kruskal–Wallis test with a post-hoc comparison of mean ranks. Larval development time (period between larval entry into host plant and pupation), larval survivorship, and female pupal mass were calculated and analyzed by combining data from the two experiments using a general linear mixed model with treatment as a fixed effect and experiment as a random effect. Post hoc Tukey LSD was used to compare fixed effect treatment means of the response variables in a pair-wise fashion. Size differences exist between pupae of opposite sexes; only mass of female pupae is presented because female mass has been associated with fecundity (Honĕk 1993; Tammaru et al. 2002), and it eliminates bias associated with uneven sex ratios in our dataset. Analyses were conducted using Minitab 15 (Minitab, Inc.). No significant differences in plant defense or larval performance were discovered due to host plant species, so plant species was not used as a factor in the final model. For all larval performance analyses, standard residuals were investigated by plotting them. Assumptions of the linear model were met in all analyses.

Replicate experiments were conducted during sequential years at two universities to replicate treatment combinations. At Mississippi State University (MSU), three treatments (*C. cactorum* alone, *C. cactorum* with *M. prodenialis*, and *M. prodenialis* with *C. cactorum*) were separated in time due to laboratory space availability and availability of field-collected eggs (Fig. 3A). The second experiment, performed at Arkansas State University (ASU), separated four experimental treatments in space (*C. cactorum* alone, *C. cactorum* with *M. prodenialis*, *M. prodenialis* with *C. cactorum*, and *M. prodenialis* alone) (Fig. 3B). Besides the different separation of experimental treatments (temporal vs. spatial), a few additional differences existed between the two experiments: (1) different experimental locations (previously discussed), (2) the addition of T5 fluorescent grow lighting in Experiment 2 (note that both experiments had the same light regime, but Experiment 2 had more intense light), and (3) the use of neonate larvae (Experiment 1) versus unhatched egg masses (Experiment 2). Because of these factors unique to each experiment, the data analysis included “Experiment” as random effect in the linear model. Significant differences in the treatments (fixed effects) are considered robust given that differences in experimental conditions between the replicate studies added variation (Table 1).

**Table 1:** General linear mixed model results for larval performance measures. Despite a significant difference between the two experiments, the influence of our intended experimental treatments ranged from 4× to 40× stronger (based on F-statistics) than that of temporal replication.

Source	Variable type	DF	Adjusted sum of squares	Adjusted mean squares	F	*P*
**Average larval period**						
Treatment	Fixed	3	597,440	199,147	333.5	<0.001
Experiment	Random	1	97,4936	97,4936	8.3	<0.005
Error		167	99,731	597,597		
Total		171				
**Survivorship to pupation**						
Treatment	Fixed	3	85,126	28,375	31.5	<0.001
Experiment	Random	1	97,6837	97,6837	7.6	<0.006
Error		221	597,901	597,901		
Total		225				
**Average female pupal mass**						
Treatment	Fixed	3	0.4592	0.1531	53.4	<0.001
Experiment	Random	1	0.0384	0.0384	13.4	<0.001
Error		154	0.4413	0.0029		
Total		158				

## Results

### Differential defense

**Figure 4:**
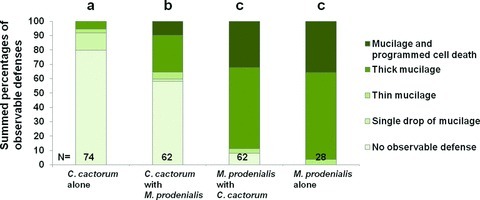
Host plant defense responses resulting from the four treatments applied. Percentage of plants defending at each of the described observable defense levels is shown. Data were analyzed with a Kruskal–Wallis test followed by a least significant difference in ranks test to determine pair-wise differences. Different letters above bars indicate significant differences between treatments.

**Figure 5:**
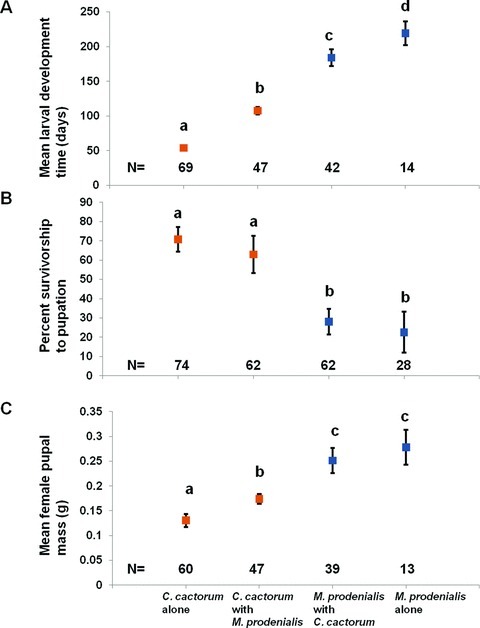
Larval performance responses to the four treatments applied. (A) Average larval development time, (B) percent survivorship to pupation, and (C) mean female pupal mass for each of the experimental treatments. Orange squares denote *Cactoblastis cactorum* and blue squares denote *Melitara prodenialis* larval performance responses. Error bars are 95% confidence intervals; different letters above mean points indicate significant differences at *P* < 0.05. Data were analyzed with a general linear mixed model with herbivore treatment as a fixed effect and experiment as a random effect. Pair-wise differences were determined with a Tukey LSD. Size differences exist between pupae of opposite sexes; only mass of female pupae is presented to eliminate bias associated with uneven sex ratios.

When reared separately, host plants defended to a much greater degree against the coevolved *M. prodenialis* than the newly-associated *C. cactorum* (Kruskal–Wallis H = 117.2, *P* < 0.001, df = 3; Fig. 4). Larval development time was fourfold longer for *M. prodenialis* than for *C. cactorum* (F_3,167_= 333.5, *P* < 0.001; Fig. 5A; Table 1), mean survivorship was threefold lower for *M. prodenialis* than for *C. cactorum* (F_3,221_= 31.5, *P* < 0.001; Fig. 5B; Table 1), and mean female pupal mass was twofold higher for *M. prodenialis* than for *C. cactorum* (F_3,154_= 53.4, *P* < 0.001; Fig. 5C; Table 1).

We have evidence that the long *M. prodenialis* larval period (Fig. 5A) as well as the large female pupal mass (Fig. 5C) was in response to defenses produced by the host plants as opposed to simply species differences between *M. prodenialis* and *C. cactorum*. We conducted a trial in which *M. prodenialis* larvae were divided between two containers and fed synthetic diet (i.e., a non-defending food source; see Marti et al. (2008) for synthetic diet recipe). For *M. prodenialis* reared on synthetic diet, the average larval period was more than threefold shorter than on defending host plants (2-sample *t*-test: *t*=–30.09, df = 90, *P* < 0.001; diet mean development time = 61.4 days ± 1.8 SE, *n*= 23 caterpillars in two cages; host plant development time = 212.1 days ± 4.7 SE, *n*= 74 caterpillars in 13 cages). The average female pupal mass was 1.4-fold smaller for larvae fed on synthetic diet than on defending host plant (2-sample *t*-test: *t*=–3.62, df = 17, *P*= 0.002; diet mean female pupal mass = 0.189g ± 0.018 SE, *n*= 9 caterpillars in two cages; host plant mean female pupal mass = 0.271g ± 0.017 SE, *n*= 36 caterpillars in 13 cages). The larval development times and pupal masses of diet-reared *M. prodenialis* are more similar to *C. cactorum* than to *M. prodenialis* individuals reared on defending hosts.

### Defense signaling

When *C. cactorum* was reared together with *M. prodenialis*, defense signaling was exhibited by plants fed upon by *C. cactorum* (Kruskal-Wallis H = 117.2, *P* < 0.001, df = 3; Fig. 4). Just over 5% of plants defended with thick mucilage when *C. cactorum* was reared alone, but over 35% of plants defended with thick mucilage and/or programmed cell death against *C. cactorum* when this species was reared in the same room as *M. prodenialis* (Fig. 4). *Cactoblastis cactorum* reared with *M. prodenialis* experienced decreased performance based on larval development time when compared with *C. cactorum* reared alone (F_3,167_= 333.5, *P* < 0.001; Fig. 5A; Table 1). Female pupae of *C. cactorum* reared with *M. prodenialis* weighed more than female pupae of *C. cactorum* reared alone (F_3,154_= 53.4, *P* < 0.001; Fig. 5C; Table 1). Though survivorship between the two *C. cactorum* treatments was not significantly different in the laboratory (Tukey LSD pair-wise *P*= 0.23; Fig. 5B), a twofold increase in larval development time would likely contribute to survivorship differences in nature. Thus, our data show that *Opuntia* defended more often and more strongly against *C. cactorum* when in the presence of other *Opuntia* plants eaten by *M. prodenialis*.

## Discussion

### Differential defense

Our experimental results are in agreement with recent findings that coevolved host–herbivore interactions provide bottom-up control on native herbivores, but may allow for outbreaks of newly-associated invasive insect species (Parker et al. 2006; Gandhi and Herms 2009; Raupp et al. 2010; Desurmont et al. 2011). During their coevolution, North American *Opuntia* likely evolved the ability to recognize and defend against *M. prodenialis*, whereas *C. cactorum* represents a novel threat, against which *Opuntia* does not or cannot defend (Pimentel 1963). Without knowledge of their different evolutionary histories, similar plant responses to larvae would be expected as both moth species appear to have identical feeding strategies (Neunzig 1997; Baker and Stiling 2009). In contrast with this expectation, our results suggest coevolution has a stronger influence than convergent feeding habits.

The signals involved in recognition and defense in this study system are yet unknown, but in various systems inducible defense response has been shown to be initiated by chemical recognition of a specific herbivore (Alborn et al. 1997; Paré et al. 1998; Felton and Tumlinson 2008; Wu and Baldwin 2009; Wu and Baldwin 2010). Some insects have been found to repress or inhibit defenses, in some cases through the upregulation of conflicting pathways in the host plant (Musser et al. 2002); this could be a possible mechanism used by *C. cactorum* in this system. Alternatively, North American *Opuntia* could be naïve to the feeding of *C. cactorum* (Gandhi and Herms 2009; Desurmont et al. 2011) if *C. cactorum* lacks the specific elicitors that cue host plant defense against *M. prodenialis*.

On non-defending food sources, larval development times for both our moth study species were similar to those of previous laboratory studies (Carlton and Kring 1994; Legaspi and Legaspi 2007; Mafokoane et al. 2007; Marti et al. 2008). When plants were defending, however, larval development times for both species increased to levels unprecedented in the literature. Pimentel (1963) observed that most successful biological control agents had previous associations only with related species or genera of host, but lacked an evolutionary history with the species controlled. Therefore, it is possible that the shared evolutionary history between *O. stricta* and *M. prodenialis* is the reason that *C. cactorum* was identified as a superior control agent for *O. stricta* over *M. prodenialis* (Dodd 1940).

### Defense signaling

Since each plant was potted individually and had no physical contact with any other plant, we deduce that plant–plant signaling is occurring for plants eaten by *C. cactorum* following detection of HIPVs released from the plants defending against *M. prodenialis* (Farmer 2001; Heil and Silva Bueno 2007; Arimura et al. 2010; Karban et al. 2010). Our results, therefore, appear to demonstrate that HIPVs initiated by native, coevolved herbivores can either induce defense in evolutionarily naïve host plant species or allow host plants to overcome novel counterdefenses of newly-associated herbivores. Given the clonal nature of *Opuntia*, plant–plant signaling in this system may have arisen as within-plant signals and as cues among kin (Heil and Karban 2010). There is an untested alternative, however. Even though plants have never been shown to directly respond to insect-derived volatile signals (Weinhold and Baldwin 2011), it is possible that selective pressures would favor this adaptation. Therefore, insect-derived volatiles are being pursued as an alternative to plant–plant signaling in this system.

Elevated defenses against *C. cactorum* resulted in significant larval performance consequences. Our results show that larval development time was extended and pupal mass of *C. cactorum* increased in plants exhibiting elevated defenses (Fig. 5). The change in larval development time indicates that host plant resistance reduces larval performance, likely from chemical defenses that limit food supply, reduce nutrient value, or interfere with hormones in the larvae (Chen 2008). Threats and suboptimal conditions in nature can create more opportunities for larvae with increased development time to succumb to predators, parasitoids, infection, starvation, and desiccation, thus reducing survivorship (Häggström and Larsson 1995; Benry and Denne 1997; Coley et al. 2006; Cornelissen and Stiling 2006). Moreover, our data are in agreement with previous findings that show longer development time in most insects is correlated with increased body size (Berger et al. 2006; Coley et al. 2006) (Fig. 5). Large body size also has been linked to increased susceptibility to predators, so it is possible that reductions in survivorship could be compounded in nature due to the combined larger body size and extended larval period (Berger et al. 2006). Therefore, responses resulting from defense-induced plants have the potential to decrease the survivorship and reproductive output of *C. cactorum* to a greater degree in nature than in the laboratory. In most insect species, however, large females are able to produce greater numbers of healthier offspring (Honĕk 1993; Tammaru et al. 2002), indicating possible trade-offs to increased body size. Field studies are needed to assess how induced defenses in nature could influence the population dynamics of *C. cactorum*, particularly with regard to the overall trade-off between increased pupal size and fitness.

Our discovery that putative HIPVs initiated by a native, coevolved herbivore can induce defense against a newly-associated insect pest suggests that defense signaling may have important applications for controlling *C. cactorum*, as has been suggested for other plant pest species (Thaler 1999; Khan et al. 2008; Gurr and Kvedaras 2010; Orre et al. 2010; Simpson et al. 2011). Identification and testing of HIPVs involved in defense signaling of *Opuntia* is currently underway. Investigations of the possible uses of *M. prodenialis* to reduce negative impacts of *C. cactorum* on native *Opuntia* individuals and populations will need to be tested in field conditions. Further exploration of defense signaling in relation to species invasions and additional research on the influence of evolutionary history on plant–herbivore interactions are greatly needed.
